# Supplementary material for: The Role of Bacteriophage-Derived Small RNA Molecules in Bacterial and Phage Interactions
Source: Viruses. 2025 Jun 10;17(6):834. doi: 10.3390/v17060834 (PMC12197685; doi:10.3390/v17060834)
Supplement: Supplementary file 1 [file viruses-17-00834-s001.zip › viruses-3676594-supplementary.pdf]

Supplementary Table S1. Phage-derived molecules that have been identified; however, their specific functional roles within the cell remain undefined.

sRNA name	Host	Phage	Size (nt)	Potential function	References
LPR2	*Escherichia coli*	λ	~63	Associated with the lytic cycle	[17]
6S	*Escherichia coli*	λ	~194 and ~121	Associated with the lytic cycle	[17]
PAK_P4as06 RNA	*Pseudomonas aeruginosa*	PAK_P4	200	Unknown	[18]
PAK_P4as07 RNA	*Pseudomonas aeruginosa*	PAK_P4	200	Unknown	[18]
PAK_P4as14 RNA	*Pseudomonas aeruginosa*	PAK_P4	200	Unknown	[18]
PAK_P4as16 RNA	*Pseudomonas aeruginosa*	PAK_P4	200	Unknown	[18]
STnc6000	*Salmonella enterica* serovar Typhimurium D23580	BTP1	-	Unknown	[27]
STnc6080	*Salmonella enterica* serovar Typhimurium D23580	BTP1	-	Unknown	[27]
STnc6010	*Salmonella enterica* serovar Typhimurium D23580	BTP1	-	Unknown	[27]
STnc6020	*Salmonella enterica* serovar Typhimurium D23580	BTP1	-	Unknown	[27]
STnc6030	*Salmonella enterica* serovar Typhimurium D23580	BTP1	-	Unknown	[27]
STnc6040	*Salmonella enterica* serovar Typhimurium D23580	BTP1	-	Unknown	[27]
STnc6050	*Salmonella enterica* serovar Typhimurium D23580	BTP1	-	Unknown	[27]
STnc6060	*Salmonella enterica* serovar Typhimurium D23580	BTP1	-	Unknown	[27]

STnc6070	*Salmonella enterica* serovar Typhimurium D23580	BTP1	-	Unknown	[27]
misc_1	*Yersinia enterocolitica*	φR1-37	129	Unknown	[40]
misc_2	*Yersinia enterocolitica*	φR1-37	394	Unknown	[40]
misc_3	*Yersinia enterocolitica*	φR1-37	320	Unknown	[40]
misc_5	*Yersinia enterocolitica*	φR1-37	102	Unknown	[40]
misc_6	*Yersinia enterocolitica*	φR1-37	340	Unknown	[40]
misc_7	*Yersinia enterocolitica*	φR1-37	350	Unknown	[40]
misc_8	*Yersinia enterocolitica*	φR1-37	310	Unknown	[40]
misc_9	*Yersinia enterocolitica*	φR1-37	174	Unknown	[40]
misc_10	*Yersinia enterocolitica*	φR1-37	324	Unknown	[40]
EcOnc03	*Escherichia coli* O157:H7 str. Sakai	Lambdoid prophage encoded	~54	Unknown	[45]
EcOnc04	*Escherichia coli* O157:H7 str. Sakai	Lambdoid prophage encoded	~222	Unknown	[45]
EcOnc05	*Escherichia coli* O157:H7 str. Sakai	Lambdoid prophage encoded	~222	Unknown	[45]
EcOnc06	*Escherichia coli* O157:H7 str. Sakai	Lambdoid prophage encoded	~177	Unknown	[45]
EcOnc08/21/42	*Escherichia coli* O157:H7 str. Sakai	Lambdoid prophage encoded	~271	Unknown	[45]
EcOnc13	*Escherichia coli* O157:H7 str. Sakai	Lambdoid prophage encoded	~187	Unknown	[45]
EcOnc18	*Escherichia coli* O157:H7 str. Sakai	Lambdoid prophage encoded	~37	Unknown	[45]
EcOnc22	*Escherichia coli* O157:H7 str. Sakai	Lambdoid prophage encoded	~85	Unknown	[45]
EcOnc27	*Escherichia coli* O157:H7 str. Sakai	Lambdoid prophage encoded	~247/235	Unknown	[45]
EcOnc32	*Escherichia coli* O157:H7 str. Sakai	Lambdoid prophage encoded	~112	Unknown	[45]

EcOnc33	*Escherichia coli* O157:H7 str. Sakai	Lambdoid prophage encoded	~71	Unknown	[45]
EcOnc50	*Escherichia coli* O157:H7 str. Sakai	Lambdoid prophage encoded	~78	Unknown	[45]
EcOnc38	*Escherichia coli* O157:H7 str. Sakai	Lambdoid prophage encoded	~256	Unknown	[45]
